# Effects of strength training on osteogenic differentiation and bone strength in aging female Wistar rats

**DOI:** 10.1038/srep42878

**Published:** 2017-02-17

**Authors:** Monique Patricio Singulani, Camila Tami Stringhetta-Garcia, Leandro Figueiredo Santos, Samuel Rodrigues Lourenço Morais, Mário Jefferson Quirino Louzada, Sandra Helena Penha Oliveira, Antonio Hernandes Chaves Neto, Rita Cássia Menegati Dornelles

**Affiliations:** 1 Programa Multicêntrico de Pós-Graduação em Ciências Fisiológicas, Unesp-SBFis, Brasil; 2 Univ Estadual Paulista (Unesp), Faculdade de Medicina Veterinária, Departamento de Apoio, Produção e Saúde Animal, Araçatuba, 16050-680, Brasil; 3 Univ Estadual Paulista (Unesp), Faculdade de Odontologia, Departamento de Ciências Básicas, Araçatuba, 16018-805, Brasil

## Abstract

The effects of strength training (ST) on the mechanical bone strength and osteogenic differentiation of bone marrow mesenchymal stromal cells (BMSCs) from adult, aged and exercised aged rats were determined. The exercised aged animals displayed higher values of areal bone mineral density, compression test, alkaline phosphatase activity (ALP) and biological mineralization, while oil red O staining for adipocytes was lower. ST increased gene expression of runt-related transcription factor 2 (*Runx2*), osterix (*Osx*) as well as bone matrix protein expression, and reduced expression of peroxisome proliferator-activated receptor gamma (*Pparγ*). The production of pro-inflammatory cytokine tumor necrosis factor alpha (TNF-α) was lower in BMSCs of the aged exercised group. The ST practice was able to improve the bone mechanical properties in aged female rats, increasing the potential for osteogenic differentiation of BMSCs, reducing the adipogenic differentiation and pro-inflammatory cytokine level. In summary, the data achieved in this study showed that strength training triggers physiological responses that result in changes in the bone microenvironment and bring benefits to biomechanical parameters of bone tissue, which could reduce the risk of fractures during senescent.

Osteoporotic fracture is considered a major worldwide epidemic, resulting in serious increased morbidity and mortality in older women^1^,^2^. In this phase of life, there is a negative balance between the volumes of bone resorbed and formed during remodeling, which accelerates bone mass loss, leading to architectural deterioration and decreased bone strength^1^. Physical exercise practice, combined or not with pharmacological treatment, has been shown to prevent osteoporosis and osteoporotic fractures in postmenopausal women^2^ and aged female rats^3^.

The etiopathogenesis of aging-associated osteoporosis has been linked to changes in the number of mesenchymal stem cells, capacity for osteogenic differentiation^4^ and increased pro-inflammatory cytokine levels in bone marrow^5^. It leads to a shift in the bone microenvironment resulting in a decrease in bone mass with increasing age. In addition, physical inactivity in older individuals contributes to increase the differentiation potential of the adipocyte lineage, decreasing bone quality^4^.

Mechanical stimulation of the bone is able to induce differentiation of mechanosensitive mesenchymal stem cells into osteoblasts^6^,^7^, which in turn activates the transcription factor for regulating osteogenic expression, osteoblast maturation and bone formation^8^,^9^. Some transcription factors, like runt-related transcription factor 2 (Runx2) and Osterix (Osx), are related with increases in mesenchymal stem cells under mechanical strain. Runx2 is described as a positive regulator of osteoblast differentiation that can upregulate the expression of bone matrix protein genes, including collagen, type I, alpha 1 (Col1a1), osteopontin (Opn), bone sialoprotein (Bsp) and osteocalcin (Ocn)^10^. Osx has been shown to act as a downstream factor of Runx2 in the osteoblast maturation and enhance the proliferation and osteogenic lineage commitment of the bone marrow stromal cells^11^. Previously, it has been shown that mechanical loading exercise reduces the risk of bone loss, while it increases bone mass and bone remodeling processes^12^,^13^, bringing benefits to the musculoskeletal system of postmenopausal women^2^ and female rodents^3^,^6^. However, there is little knowledge about mechanisms by which exercise reduces or reverses bone loss in aged females. In view of the well-known role of mesenchymal stromal cells in osteogenesis, a better understanding about age-related changes in osteogenic differentiation resulting from mechanical stimulation is essential to unravel how mechanical strain is able to control primary osteoporosis.

Therefore, the objective of this study was to determine the effects of strength training (ST) performed *in vivo* on the physical properties of bone and potential osteogenic differentiation of bone marrow mesenchymal stromal cells (BMSCs) isolated from aged female rats. Our hypothesis is that stimulation provided by ST performed *in vivo* during senescent period, in periestropause, might represent a way to prevent the characteristic bone loss of this period of life.

## Results

### Body weight and the carrying capacity

There was a significant difference between the initial and final body weight of adult and aged animals (*p* < 0.001 and *p* = 0.030), but ST practice did not influence the final body weight of exercised aged animals in comparison to non-exercised aged animals (*p* = 0.782) (Table 1). All animals, both adult and aged, displayed a significant increase in the initial maximum voluntary carrying capacity (MVCC) (*p* < 0.001), however, when MVCC expressed as relative value to the body weight, this increase not is evidenced. The aged group displayed decreased final (76.68%) MVCC in comparison to initial (85.53%) MVCC (*p* < 0.05). At the beginning of ST, exercised aged animals had a carrying capacity value of 81.69% of their body weight. At the end of ST, the same animals had a carrying capacity value of 128.81% of their body weight (Table 1).

### Biomechanical properties of bone

In order to evaluate whether ST could influence the bone strength, we assessed areal bone mineral density (aBMD) by dual-energy X-ray absorptiometry (DEXA) and biomechanical compression testing measured in left femurs.

**Figure 1:**
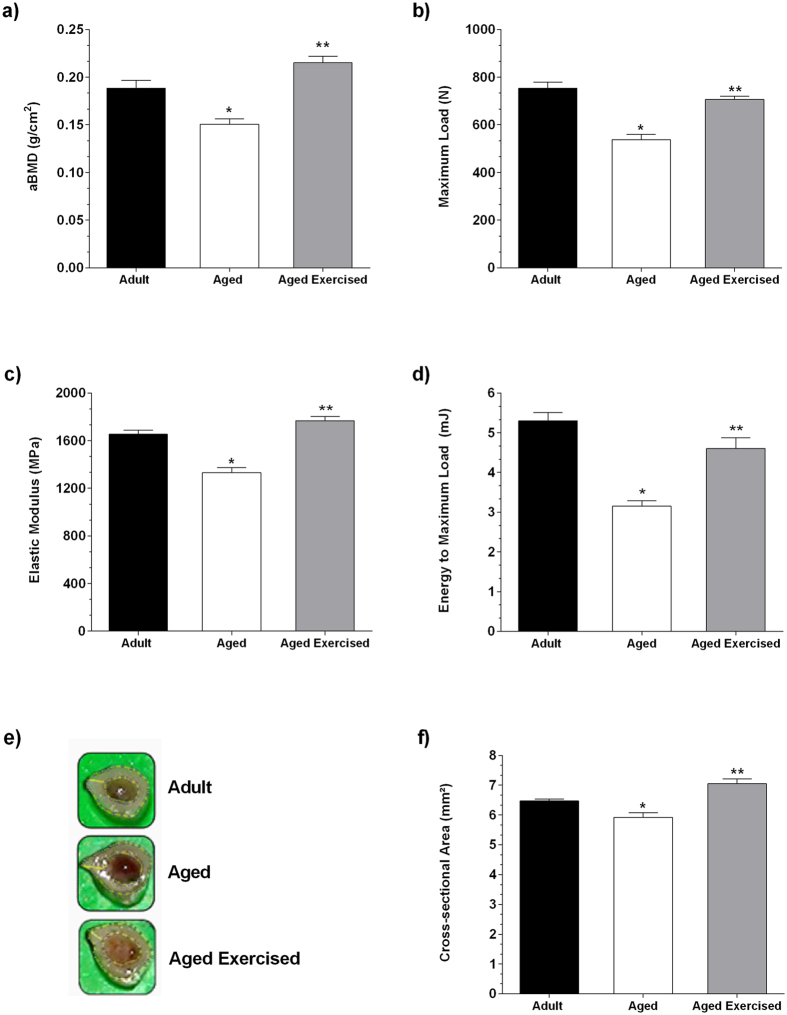
BMD and cross-sectional of femur after strength training in aging. Strength training effect on areal bone mineral density (aBMD) measured by dual-energy X-ray absorptiometry (**a**), maximum load (**b**), elastic modulus (**c**) and energy to maximum load (**d**) measured by compression test in femurs from adult and aged female rats. Images of the cross-sectional area were photographed (**e**), and cross-sectional area was measured by ImageJ software in femurs from adult and aged female rats (**f**). Data were expressed as means ± standard error (SEM) and examined using the unpaired Student’s *t*-test. *p < 0.05 vs. adult, **p < 0.05 vs. aged rats.

Aged rats presented decreased aBMD (*p* = 0.001), maximum load (*p* < 0.001), elastic modulus (*p* = 0.001), energy to maximum load (*p* < 0.001) and cross-sectional areas of bone specimen (*p* = 0.007) when compared to adult rats (Fig. 1a–f). After the ST, it was verified an increase in aBMD (*p* < 0.001), maximum load (*p* < 0.001), elastic modulus (*p* < 0.001), energy to maximum load (*p* < 0.001) and cross-sectional areas (*p* < 0.001) in the exercised aged animals when compared to non-exercised aged animals (Fig. 1a–f).

### Proliferative rate and alkaline phosphatase activity in BMSCs

**Figure 2:**
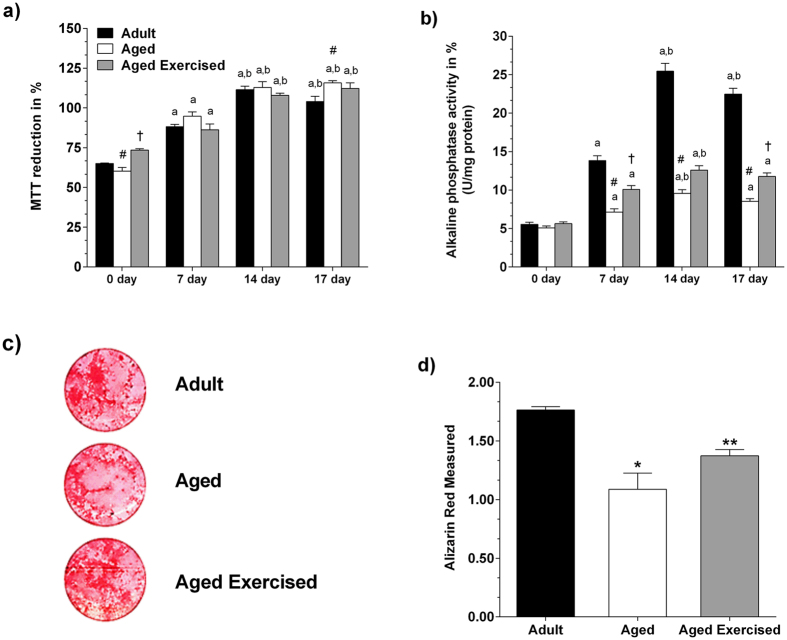
MTT and ALP after strength training in aging. Strength training effect on level of 3-[4,5-dimethylthiazole-2-yl]-2,5-diphenyltetrazolium bromide (MTT) reduction (**a**) and alkaline phosphatase (ALP) activity (**b**) in the bone marrow mesenchymal stromal cells (BMSCs) from adult and aged female rats after osteogenic induction. Results were presented as values multiplied by one hundred. Calcium deposits by alizarin red (**c**) and alizarin red measured (**d**) on day 21 after osteogenic induction. Data were expressed as means ± standard error (SEM). Statistical analysis of data from MTT and ALP assays were done using one-way repeated measures ANOVA followed by Tukey’s *post-hoc* test, to compare the differences across test days (^a^*p* < 0.05 *vs.* 0 day in the same group, ^b^*p* < 0.05 *vs.* 7 day in the same group), while the one-way ANOVA, followed by Bonferroni’s *post-hoc* test, was used to compare the differences between the groups in the same day (^#^*p* < 0.05 *vs.* adult in the same day and ^†^*p* < 0.05 *vs.* aged in the same day). The results from alizarin red measurement were analyzed statistically using Student’s *t*-test (*p < 0.05 *vs.* adult, **p < 0.05 *vs.* aged rats).

The proliferative rate assessed by the 3-[4,5-dimethylthiazol-2-yl]-2,5-diphenyltetrazolium bromide (MTT) reduction assay and alkaline phosphatase (ALP) activity were analyzed in BMSCs after 0, 7, 14 and 17 days of osteogenic differentiation. A time-dependent increase in proliferation rate was observed in all experimental groups, indicating the ability to maintain proliferation during long-term culture. The effect of the donor’s age on MTT reduction in BMSCs was only observed at 17 days when the culture from the aged rat cells eventually showed larger reduction (*p* = 0.026) in MTT into formazan crystals compared to the adult group, due the differentiation process of the BMSCs (Fig. 2a). However, physical activity did not alter this parameter because there was no significant difference between the cells from exercised aged and non-exercised aged animals (Fig. 2a).

The ALP activity (Fig. 2b) presented an increasing trend until a peak at day 14, with further decreasing trend, for all experimental groups, which is a characteristic pattern for this culture model. Regarding the differences between experimental groups, ALP activity was significantly lower in the aged groups, compared to the adult group at day 7 (*p* < 0.001), at day 14 (*p* < 0.001) and at day 17 (*p* < 0.001) (Fig. 2b). However, ST was able to generate a significant increase in the ALP activity rate in the exercised aged group, compared to the non-exercised aged group at day 7 (*p* = 0.004), and at day 17 (*p* = 0.018) (Fig. 2b).

### Mineralization in BMSCs

To verify whether ST-induced increase in ALP activity was accompanied by improved mineralization, we assessed the Alizarin red staining at 21 days (Fig. 2c). Alizarin Red staining resulted in marked differences between adult and aged groups (*p* < 0.001) (Fig. 2c,d). When compared with the non-exercised aged group, the quantity of calcium deposition in the exercised aged group was significantly higher (*p* < 0.001), showing a 21% increase in mineralization (Fig. 2c,d).

### Effect of ST practice in the osteogenic and adipogenic differentiation gene expression

**Figure 3:**
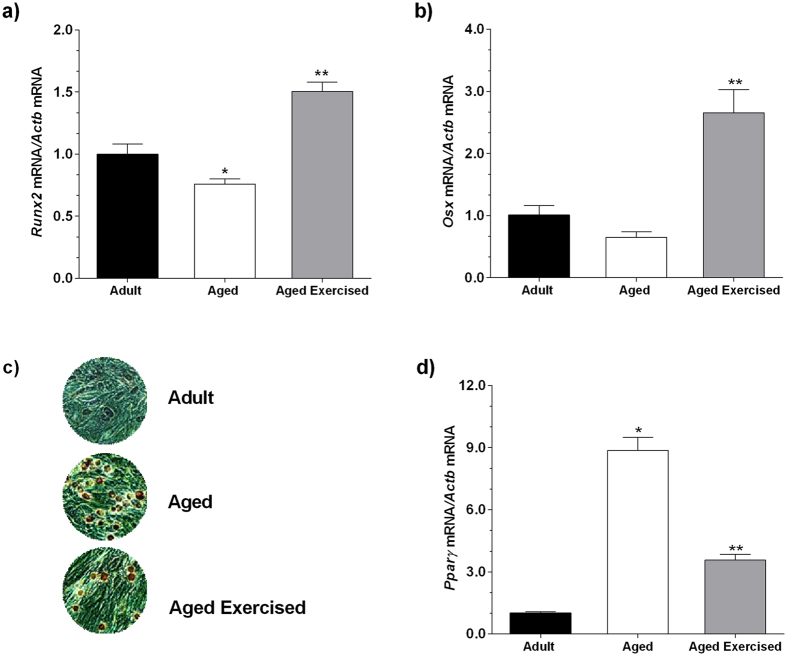
mRNA expression of the osteogenic transcription factors in the BMSCs. Strength training effect on mRNA expression of the osteogenic transcription factors runt-related transcription factor 2 (*Runx2*) (**a**), Osterix (*Osx*) (**b**) by quantitative real-time PCR (qRT-PCR); lipid content (**c**) by oil red O staining and mRNA expression of transcription factor of peroxisome proliferator-activated receptor gamma (*Pparγ*) (**d**), by qRT-PCR in the bone marrow mesenchymal stromal cells (BMSCs) from adult and aged female rats on day 14 after osteogenic induction. The reactions were carried out in duplicate, and the normalized values were subjected to the fold change. The samples were normalized to the adult rat group. Data were expressed as means ± standard error (SEM) and examined using the unpaired Student’s *t*-test. *p < 0.05 vs. adult, **p < 0.05 vs. aged rats.

We examined the transcription factors involved in the osteoblast differentiation gene expression at a time interval of 14 days. *Runx2* expression was considerably increased in the adult group compared with the aged group (*p* = 0.040) (Fig. 3a,b). However, ST performed in the aging rats has provided an increase in the levels of *Runx2 (p* < 0.001) (Fig. 3a) and *Osx (p* = 0.035) (Fig. 3b), when compared with the aged group.

We also examined the peroxisome proliferator-activated receptor gamma (*Pparγ*) transcription factor involved in the adipogenesis differentiation gene expression, accomplished at a time interval of 14 days. *Pparγ* expression was significantly increased in the non-exercised aged group compared with the adult (*p* = 0.006) and exercised aged groups (*p* = 0.017) (Fig. 3d). At the 21^st^ day, the cells were stained with Oil Red O solution to identify lipid droplets. This staining revealed that the lipid accumulation in BMSCs was higher in the non-exercised aged group compared with the adult and exercised aged groups (Fig. 3c), contributing to the results obtained by *Pparγ* expression (Fig. 3d).

### ST and extracellular matrix proteins expression

**Figure 4:**
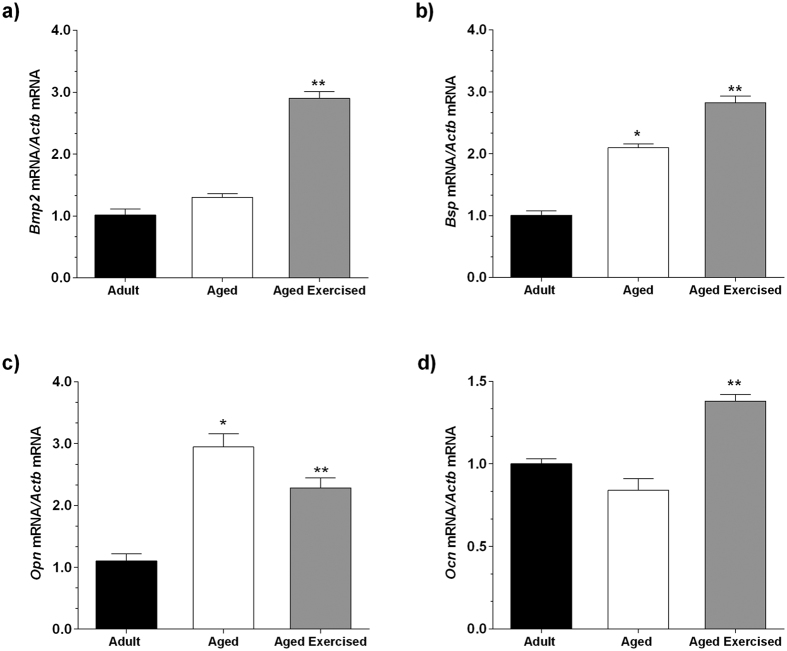
mRNA expression of the bone matrix proteins in bone cells. Strength training effect on the mRNA expression of the bone matrix proteins bone morphogenic protein 2 (*Bmp2*) (**a**), integrin-binding sialoprotein (*Bsp*) (**b**), osteopontin (*Opn*) (**c**), and osteocalcin (*Ocn*) (**d**) by quantitative real-time PCR (qRT-PCR) in bone marrow mesenchymal stromal cells (BMSCs) from adult and aged female rats on day 14 after osteogenic induction. The reactions were carried out in duplicate, and the normalized values were subjected to the fold change. The samples were normalized to the adult rat group. Data were expressed as means ± standard error (SEM) and examined using the unpaired Student’s *t*-test. *p < 0.05 vs. adult, **p < 0.05 vs. aged rats.

To investigate the transcription factors involved in quality of extracellular matrix proteins, quantitative real-time PCR (qRT-PCR) analysis was performed. Bone morphogenic protein 2 (*Bmp2*) expression was significantly higher in the exercised aged compared with the non-exercised aged group (*p* < 0.001) (Fig. 4a), as well as for the *Bsp* expression (*p* = 0.027) (Fig. 4b). However, the aged group showed a considerable increase in *Opn* expression when compared with the adult group (Fig. 4c) demonstrating a delay in expression of osteogenic markers in the aged group. The results also indicated that ST increased the *Ocn* expression when compared with the aged group (*p* = 0.021) (Fig. 4d).

### The production of pro-inflammatory cytokines after ST

**Figure 5:**
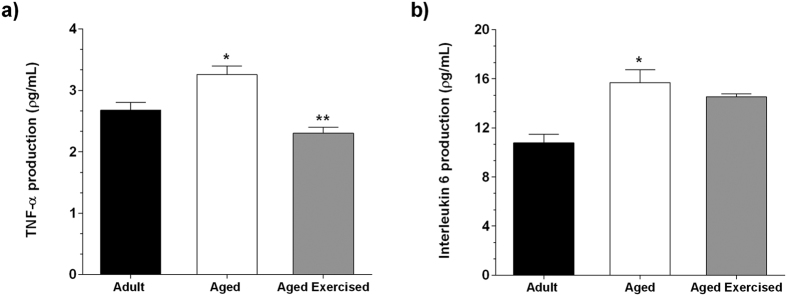
TNF-α and IL-6day 14 after osteogenic induction. Strength training effect on concentration of tumor necrosis factor alpha (TNF-α) (**a**) and interleukin-6 (IL-6) (**b**) in the bone marrow mesenchymal stromal cells (BMSCs) supernatant from adult and aged female rats on day 14 after osteogenic induction. The cytokine concentrations were measured by Enzyme-Linked Immunosorbent Assay (ELISA). Data were expressed as means ± standard error (SEM) and examined using the unpaired Student’s *t*-test. *p < 0.05 vs. adult, **p < 0.05 vs. aged rats.

The production of tumor necrosis factor alpha (TNF-α) (Fig. 5a) and interleukin-6 (IL-6) (Fig. 5b) in the BMSCs supernatant, evaluated by ELISA assay, was significantly higher in the aged group when compared with the adult group (*p* = 0.038 and *p* = 0.017). However, the exercised aged groups showed decreased production of TNF-α in comparison with the non-exercised aged groups (Fig. 5a). We also observed a decline of IL-6 in these animals, although it was not significant (Fig. 5b).

## Discussion

Our results evidenced that the progressive load exercise program had a stimulatory effect in the bone of aged female rats. Our observations might be explained, at least in part, by an increase in both osteoblastic commitment and bone marrow mesenchymal stromal cells differentiation, effects that were probably due to the enhanced expression of transcription factors and matrix proteins that are important for bone metabolism. This study presents interesting results about the long-lasting effects of physical exercise during aging on biological mechanisms at the cellular level and the mechanical properties in bone during the periestropause period, when changes in bone homeostasis are observed, resulting in major probability of fractures by fragility.

In the present study, we demonstrated that progressive overload imposed in aged rats during ST significantly increased aBMD and was positively associated with maintained bone homeostasis. The mechanical integrity of bone, determined in this study by analysis of the maximum load, elastic modulus and energy to maximum load, showed that bone deformation threshold provided by the training program favored adaptations for strength and resistance, given the increase in aBMD and cross-sectional bone area from exercised aged rat. Furthermore, it is likely that the expression of growth factors and the increase of bone matrix production favored osteogenesis, consequently improving bone structural and mechanical properties in exercised aged animals. We believe that these findings reflect the intensity of the ST program, i.e., using low repetition/high overload^3^,^13^,^14^, in which aged rats displayed a MVCC value, expressed as relative value to the body weight, of 128.81% in comparison to the values obtained previous to ST program practice (Table 1). In addition, it is important to consider that the length of the training period (16 weeks) contributed to the significant differences observed in biomechanical properties. This means that even when applying high impact/high intensity programs, exercise frequency and its maintenance play a key role in bone adaptation^15^, such as demonstrated in this study.

Studies have shown that the osteogenic differentiation potential of cultured BMSCs changes according with the age of the donor and the treatment applied both *in vivo* and *in vitro*, by means of cellular subculture^6^,^7^. Others previous reports have shown that BMSCs cultures subjected to vibrations exhibited an increase in their intrinsic potential for cellular activation and osteoblastic differentiation compared with those that were not vibrated^8^. Therefore, in order to obtain a cell population with a characteristic stromal microenvironment phenotype, we used the primary culture of BMCSs from aged rats that were subjected to ST compared with primary culture from aged rats did not undergo ST. We analyzed whether ST practice regulated biological mechanisms at the cellular level in aged rats. BMSCs *in vitro* should present a characteristic phenotype, once again because the pool of cells present in bone marrow are mesenchymal stem cells^16^ directly involved in stromal environment maturation^17^. This study showed that the biological characteristics of BMSCs exhibited sustained continuous growth and proliferation ability, which was maintained during long-term culture. Although cell proliferation was observed during the osteogenesis in all experimental groups, osteoblast phenotype maturation signals, like up regulation of ALP, an essential marker for matrix mineralization^18^, were lower in the aged groups, the observed. Nevertheless, the exercised aged group presented higher ALP activity when compared to the non-exercised aged group, reflecting the extracellular matrix maturation process^19^ under the influence of ST. These markers have been described to decrease with donor age, and this has been established both in human mesenchymal stem cells (hMSC) isolated from the femoral heads of aged patients^20^ and the mesenchymal stem cell isolated from rats of various ages^21^. Nevertheless, in our data, ST practice was able to promote an increase in the differentiation of BMSCs derived from aged rat into osteoblasts, resulting in a greater biological mineralization, reflecting the potential advanced cell differentiation^22^.

A study conducted by Hell *et al*.^6^ with young and adult female rats demonstrated an increase in the osteogenic differentiation of BMSCs isolated from active adult rats. The difference between the previous study and ours is that we studied animals with mature skeletons^23^ and with changes in the estrous cycle, which features an appropriate animal model for studying bone loss linked to aging. In female rats, the incidence of regular estrous cyclicity decreases progressively during aging and their estrous cycles tend to become irregular, usually with prolonged estrous and diestrous, characterizing periestropause^24^,^25^, and similar to perimenopause in women^26^,^27^, they have an increased risk of fragility fractures in this period^2^. Therefore, until recently, the effect of physical activity, specifically ST, in the osteogenic differentiation of BMSCs cultures from aged donor rats, had not been previously demonstrated.

Up to now, these findings indicate that mechanical stimulus can reverse the functional decline in the cellular microenvironment of cultured BMSCs from aged donors, resulting in increased bone formation by greater differentiation of the BMSCs into the osteoblast lineage *in vitro*. Subsequently, it was observed an increase in the expression of osteoblast-specific transcription factors, *Runx2* and *Osx* promoted by ST practice. These results suggest that ST-induced mechanical stimulus on bone drive the differentiation of mechanosensitive BMSCs. This results in a mechanical activation of bone cells that changes the bone canalicular fluid gradient and increases intracellular calcium concentration, in addition to osteogenic factors and bone matrix production^12^,^13^. The evaluation of *Pparγ* expression, a key regulator of adipocyte differentiation, confirmed that the mechanical stimulus directly increased the differentiation and bone formation and adversely altered the marrow adiposity, confirmed by decreased expression of *Pparγ* in the BMSCs of aged rats that performed ST. In ‘this study, the exercised aged group presented higher osteogenic potential due to an increased expression of *Runx2* and *Osx*, leading to the differentiation of BMSCs away from adipocytes and towards osteoblasts.

These results highlight the importance of the *Runx2* and *Osx* in directly stimulating the transcription of important osteoblast-associated proteins that support proliferation, matrix formation and mineralization^16^, such as *Bmp2, Bsp, Opn*, and *Ocn*^10^,^11^. Thus, our results showed that the bone responds to mechanical stimulus with increased expression of matrix proteins, such as *Bmp2* and *Bsp*, both necessary for the initiation of bone mineralization^28^. The expression of the non-collagenous protein *Opn* is necessary to initiate crystal formation and prevent the premature precipitation of calcium phosphate crystals that do not have the well-coordinated structure of hydroxyapatite^28^,^29^. Moreover, *Opn* may facilitate the binding of osteoclasts, favoring bone resorption^28^. Thus, considering that osteoclast activity increases with age^30^, the increased expression of *Opn* in the aged group, suggests a prevalence of the resorptive phenotype. Another protein, *Ocn*, which acts as a chemo-attractant for the recruitment and differentiation of the osteoclast progenitors and regulates the quantity of mineral deposition^28^, was significantly expressed in the exercised aged group. These results show that ST acts positively in bone modulation even under the influence of aging, when there is a poor quality of extracellular matrix due to an increase in bone resorption in comparison to bone formation^30^.

As a final point, we evaluated pro-inflammatory cytokine levels, such as TNFα and IL-6, which present negative effects on osteoblast differentiation, favoring elevated bone-resorbing activity^5^. In this study, we evaluated the physiological aging female rat to study primary osteoporosis. In women, osteoporosis, most often, is related with estrogen reduction, similar to what happens in aged female rats^3^,^31^. As the levels of estrogen decrease, the production of TNFα and IL-6 increase and play an important role in the pathogenesis of inflammatory bone loss through stimulation of bone resorption and inhibition of osteoblastic bone formation^32^. In this study, the exercises performed by aged female rats stimulated osteogenic potential by increasing the expression of osteoblast-specific transcription factors, decreasing expression of *Pparγ,* and decreasing the production of TNF-α. These results show that ST triggers a series of physiological responses involving changes in the bone microenvironment, benefiting biomechanical parameters of bone tissue in aged female rats.

Taken together, the *in vivo* and *in vitro* analyses demonstrated the positive effects of exercise during aging. We found that ST improves mechanical competence of the bone *in vivo* in exercised aged rats by modulating processes such as differentiation and up regulation of osteoblast phenotypic marker genes, while simultaneously suppressing adipocyte expression and pro-inflammatory cytokine production. These processes promote biological mineralization *in vitro*. These results add new information about molecular and cellular mechanisms underlying the osteogenic differentiation in the aging and provide good basis for preclinical studies.

## Methods

### Animals

Animal procedures were approved by Ethics Committee for Research Involving Animals of the University Estadual Paulista (CEUA: 00462/2012) and complied with the Guide for Care and Use of Laboratory Animals. Multiparous female rats (*Rattus norvergicus albinus*) at ages 5 (adult group) and 17 months old (aged group), were housed in a controlled room. Estrous cycle was checked through vaginal smears taken daily to confirm regularity in the cycle of the adult rats, and irregularity in the cycle of the aged females, characterizing the periestropause period^24^,^25^,^27^. Only adult animals (n = 10) with regular cycles and aged animals (n = 20) with irregular cycles were included in this study. Aged rats were randomly allocated into two groups: aged animals (n = 10) and exercised aged animals (n = 10). The ST in exercised aged group was carried out by climbing sessions on a ladder during 16 weeks. Adult animals were euthanized at 9 months old, and aged animals at 21 months old, always seventy-two hours after the last ST session. Adult rat group was used as a reference to compare with aged rat, since a peak in bone mass occurs around 9th month of life^23^. Both femurs from all experimental groups were quickly collected for further analysis.

### Strength training protocol and MVCC

Aged rats completed the ST program^33^, by performing climbing sessions on a ladder (1.1 × 0.18 m, 2-cm grid, 80° incline – engineered by department of maintenance Unesp, SP, Brazil). Animals underwent 1 week of acclimation performed without overload. Seventy-two hours after the last acclimated session, the initial MVCC of each animal was obtained. A load apparatus (Plastic tube, BD Biosciences^®^, MA, USA) containing steel balls (Cabana S/A, SP, Brazil) corresponding to 75% of the body mass was secured to the proximal portion of the rat’s tail with a self-adhesive foam strip (Missner & Missner Ltda, SC, Brazil). The rats performed repeated climbs with the load apparatus, resting 5 min between each climb. Then, 30 g of load was added to the load apparatus, and a new repetition was performed until climbing failure. The value of the load before climbing failure was considered the final MVCC. The test was performed for 15 days in a row, so that the correct load was applied in each animal. ST protocol consisted of 3 sessions per week (nonconsecutive days) for 16 weeks. Each session was composed of 6 sets of climbs, containing 48 and 62 dynamic movements (isotonic) per series. The first week of the ST protocol began with an overload corresponding to 60% of the MVCC, which was increased to 70% of the MVCC in the second week, and finally to 80% of the MVCC in the third week, and this last established load was maintained until the end of the protocol.

### Bone mineral density measurements

aBMD was measured in left femurs (10 specimens in each group) by DEXA (Lunar DPX Alpha, WI, USA). The equipment was calibrated according to manufacturer instructions, and the device software was used for measuring BMD in small animals. aBMD was calculated by dividing bone mineral content by bone area (cm^2^) using the software associated with the DEXA scanner in order to determine the aBMD (g/cm^2^).

### Biomechanical compression testing

Biomechanical properties of the left femurs were assessed by the compression test (Universal Testing Machine; DL 3000, EMIC®, PR, Brazil). All femurs were cut in the proximal third of the diaphysis (measuring 6 mm in length) using a diamond cut-off wheel (Dremel® 540; IL, USA) coupled to a portable low speed motor. The bone cortical wall thickness of the samples was photographed and processed with ImageJ software (NIH, Maryland, USA), version 1.48 v, for Windows. Cross-sectional area values were acquired by obtaining the ratio between bone length (mm) and diameter (mm^2^). The biomechanical compression test was performed with a deformation rate of 5 mm/min with standardized parameters of loaded cells to 2000 N of capacity until the bone fractured^34^. The machine crossbar load and displacement was monitored and recorded using the device software. A load versus deformation graph was created from the data and the values for maximum load (N) were obtained. The elastic modulus (MPa) and energy to maximum load (mJ) were obtained from the tension and elongation curves, by using the cross-sectional area and initial length of the samples.

### Isolation and primary culture of bone marrow-derived mesenchymal stem cells

Primary culture of BMSC was established from rights femurs, using a protocol adapted from elsewhere^35^. Bone marrow (10 specimens in each group) was flushed out using 20 mL of minimal essential medium (MEM), containing 10% fetal bovine serum (FBS), 2 mM l-glutamine, and 1% antibiotics (100 U/mL penicillin G, 100 μg/mL streptomycin and 0.3 μg/mL amphotericin B) (Sigma Aldrich™, St. Louis, MO, USA). After centrifugation, the cell suspension was filtered through a 70-μm size cell strainer and through 22 and 26-gauge needles and then seeded in wells of 24-wellplates (TPP, Switzerland) at a density of 19 × 10^5^ cells/cm^2^ and cultured at 37 °C under a 5% CO_2_ 95% air atmosphere. After 10 days, under subconfluence (80%), the cells were cultured in a proliferation medium (MEM) or osteogenic medium (MEM supplemented with 10 nM β-glycerophosphate, 50 μg/mL ascorbic acid and 10 nM dexamethasone), and subjected to experimental tests after 0, 7, 14, 17 and 21 days in culture, after the addition of osteogenic medium.

### MTT assay for cell proliferation

Cell proliferation and viability were measured by the MTT reduction assay after 0, 7, 14 and 17 days^36^ in culture. MTT reagent (Sigma-Aldrich™) was added to each sample to allow the formation of MTT formazan. The resulting formazan was reduced with ethanol (Merck, Darmstadt, Germany), and measured at 570 nm by a spectrophotometric microplate reader (Molecular Devices’, CA, USA). Cell proliferation was determined by comparing the absorbance of samples to a standard curve and the results were presented with the original values multiplied by one hundred.

### Alkaline phosphatase activity

ALP activity was determined after 0, 7, 14 and 17 days^37^ in culture using a commercial kit (Labtest Diagnóstica, MG, BRA). Enzyme activity was detected by thymolphthalein release and measured at 590 nm using a spectrophotometric microplate reader (Molecular Devices’). The specific activity was calculated by enzyme activity normalized by total protein content^38^. Results were presented multiplied by one hundred.

### Alizarin red staining

Mineralization in osteoblast cultures was determined by Alizarin Red S staining^39^ after 21 days in culture. Briefly, cells were fixed with paraformaldehyde (Merck) at 10% for 30 min, washed with distilled water, and stained with 20 mg/mL Alizarin Red S (Sigma Aldrich™) for 30 min. Stained cells were digitalized using a high resolution scanner and staining was quantified by spectrophotometric measure at 405 nm using a microplate reader (Molecular Devices’).

### Oil red O staining

Cells were stained for fat vacuoles by using oil red O (Sigma Aldrich™) staining after 21 days^40^ in culture. Cells were rinsed in PBS, fixed in 10% formaldehyde (Merck), stained in 0.3% oil red O solution (Sigma Aldrich™) and the lipid droplets of differentiated cells were then obtained from each experimental group. Images were captured using a digital camera system (Olympus DP12-2) coupled to an inverted optical microscope (Olympus CKX41-Olympus Optical CO., Ltd.; Japan).

### Total RNA isolation and gene expression

Total RNA from biological samples were isolated using Trizol reagent (Invitrogen, Life Technologies, NY, USA) after 14 days in culture. Total RNA from each sample was treated with DNAse I and reverse transcribed to complementary DNA (cDNA) using SuperScript™ II Reverse Transcriptase (Invitrogen). Expression of target genes was determined by qRT-PCR using gene-specific TaqMan probes (Applied Biosystems/Life Technologies, Paisley, UK) with Taqman® Gene Expression Master Mix on the StepOne™ Real-Time PCR System (Applied Biosystems/Life Technologies). Probes used for RT-PCR are listed: *Ocn* (Rn00566386_g1); *Opn* (Rn00563571_m1); *Bsp* (Rn00561414_m1); *Runx2* (Rn01512298_ml); *Osx* (Rn02769744_s1); *Pparg* (Rn00440945_m1) and *Bmp2* (Rn90931). The relative amount of each transcript was determined using the delta cycle threshold (ΔCt) values as previously described^41^. Changes in gene expression were calculated using relative quantitation of a target gene against endogenous, unchanged *Actb* (Rn00667869_m1) standard. Samples from BMSCs adult rats were used as a calibrator.

### Measurement of TNF-α and IL-6 production

Production of TNF-α and IL-6 was evaluated with ELISA. Briefly, supernatant medium of BMSCs was collected after 14 days in culture, centrifugated and measured using ELISA Kit to TNF-α (R&D Systems, Minneapolis, USA) and an IL-6 (R&D). Concentration of each TNF-α and IL-6 was calculated from a standard curve in pg/mL.

### Statistical analysis

Data were analyzed with Graph Pad Prism® software (CA, USA), version 6.0, for Windows. Values were presented as mean ± standard error (SEM) and significance of differences was assessed using the unpaired Student’s *t*-test. Significance level was set at 5% (*p* < 0.05) for comparisons between adult vs. aged; aged vs. exercised aged rats. Statistical analysis of data from MTT and ALP assays were done using repeated measures one-way ANOVA, followed by Tukey’s *post-hoc* test, to compare the differences across test days, while the one-way ANOVA, followed by Bonferroni’s *post-hoc* test, was used to compare the differences between the groups in the same day.

## Additional Information

**How to cite this article**: Singulani, M. P. *et al*. Effect of strength training on osteogenic differentiation and bone strength in aging female Wistar rats. *Sci. Rep.* **7**, 42878; doi: 10.1038/srep42878 (2017).

**Publisher's note:** Springer Nature remains neutral with regard to jurisdictional claims in published maps and institutional affiliations.

## Figures and Tables

**Table 1:** Body Weight and Maximum Voluntary Carrying Capacity.

	GROUPS		
	Adult (n = 10)	Aged (n = 10)	Aged Exercised (n = 10)
Initial body weight (g)	284.55 ± 5.90	327.47 ± 2.83*	348.13 ± 5.82
Initial MVCC test (g)	247.90 ± 4.79	280.10 ± 2.44*	284.40 ± 5.25
Initial MVCC test/bw (%)	87.12 ± 0.79	85.53 ± 1.23	81.69 ± 1.68%
Final body weight (g)	337.86 ± 9.20	368.67 ± 9.07*	365.29 ± 7.48
Final MVCC test (g)	283.39 ± 6.88	282.70 ± 4.70	470.55 ± 7.80**
Final MVCC test/bw (%)	83.85 ± 1.60	76.68 ± 1.74^a^	128.81 ± 4.66^a^,**

Initial body weight (bw) and maximum voluntary carrying capacity (MVCC) were measured before the experimental procedure (at 5 months old for adults and at 17 months old for aged animals). Final body weight and MVCC were measured after 16 weeks (at 9 months old for adults and at 21 months old for aged animals). The MVCC was presented as the mean of each group and refers to maximum strength capacity to perform climbing in a ladder with overload. The experimental groups were composed of ten animals each and the values are presented as mean ± SEM and examined using the unpaired Student’s *t*-test. *p < 0.05vs. adult; **p < 0.05 vs. aged; ^a^*p* < 0.05 vs. initial MVCC tests.
